# Supplementary material for: Testing microbiome associations with survival times at both the community and individual taxon levels
Source: PLoS Comput Biol. 2022 Sep 14;18(9):e1010509. doi: 10.1371/journal.pcbi.1010509 (PMC9512219; doi:10.1371/journal.pcbi.1010509)
Supplement: S1 Text — (PDF) [file pcbi.1010509.s001.pdf]

S1 Text

A large number of permutation schemes have been proposed for inference in linear models. We and others ([1, 2]) have found that the Freedman-Lane scheme both preserves type I error and optimizes test power. For example, we showed in [3] that permanovaFL, our implementation of PERMANOVA using the Freedman-Lane permutation, had a noticeable increase in power compared to adonis2, the implementation of PERMANOVA in the R package `vegan`, while still controlling type I error. We found a similar issue with the permutation scheme used in MiRKAT-S.

The test statistic used in MiRKAT-S when permutation-based inference is requested is $M^{\mathrm{T}}KM$, where M is the vector of Martingale residuals and K is the $n \times n$ distance (kernel) matrix (see [4] for more information on K). The observed value of the test statistic is compared to the permutation distribution of $(\mathbb{P}_r M)^{\mathrm{T}} K (\mathbb{P}_r M)$ for $r = 1, \cdots, R$, where $\mathbb{P}_r$ is the rth permutation matrix and R is the total number of permutation replicates. OMiSA adopted the same form of test statistic (except that K is the Euclidean distance matrix of power-transformed relative abundance data) and the same permutation scheme, and thus has the same issue that MiRKAT-S has, as demonstrated below.

One way to see the role of permutation scheme on power is to use the original MiRKAT program [5] to conduct a survival analysis by first obtaining the Martingale residuals from the Cox model, same as in MiRKAT-S, and then using the Martingale residual as a continuous outcome variable. Since the Martingale residual M_i already accounted for the effect of covariates X_i, we could use MiRKAT to fit the model

$$M_i = f(Z_i) + \epsilon_i, \tag{A1}$$

where Z_i denotes the microbiome data of all taxa from subject i and the $f(.)$ function is determined by the distance measure. Alternatively, we could fit the model

$$M_i = \beta_X X_i + f(Z_i) + \epsilon_i, \tag{A2}$$

in which inclusion of X_i seems redundant as M_i is already a residual after accounting for X_i and, in fact, the two vectors $M = (M_1, \ldots, M_n)^\mathrm{T}$ and $X = (X_1, \ldots, X_n)^\mathrm{T}$ are orthogonal. However, if permutation is conducted by permuting M_i, i.e., replacing M by $\mathbb{P}_r M$, then these two models are different: model (A1) corresponds to what Winkler et al. [2] call the "Still-White" method in their Table 2, while model (A2) generates the Freedman-Lane method. The difference is that, after permuting M_i, $\mathbb{P}_r M$ and X are no longer exactly orthogonal, but including X_i in the model ensures that $\mathbb{P}_r M$ is orthogonal to X when $f(Z_i)$ is fit *for each permutation.* In our experience, this orthogonality is the source of the power advantage enjoyed by the Freedman-Lane approach. The power advantage is especially large when there is strong confounding and when the orthogonalization has a large effect.

Comparing the test statistic of MiRKAT-S with that of MiRKAT, we find that the permutation-based MiRKAT-S corresponds to fitting model (A1) in MiRKAT. If model (A2) is fit in MiRKAT, we find an improvement in the power of MiRKAT to essentially equal the power of our approach (i.e., our adaptation of permanovaFL based on the Martingale residuals). Unfortunately, it seems difficult to implement a permutation scheme in the MiRKAT-S framework that is equivalent to fitting (A2) in MiRKAT, since the basis for inference in MiRKAT-S is a Cox model that has the survival time as the outcome; it would appear that generating replicate datasets with (censored) survival times would be required.

References

1. Anderson MJ, Legendre P. An empirical comparison of permutation methods for tests of partial regression coefficients in a linear model. Journal of statistical computation and simulation. 1999;62(3):271–303.
2. Winkler AM, Ridgway GR, Webster MA, Smith SM, Nichols TE. Permutation inference for the general linear model. Neuroimage. 2014;92:381–397.

3. Hu YJ, Satten GA. Testing hypotheses about the microbiome using the linear decomposition model (LDM). Bioinformatics. 2020;36(14):4106–4115.

4. Plantinga A, Zhan X, Zhao N, Chen J, Jenq RR, Wu MC. MiRKAT-S: a community-level test of association between the microbiota and survival times. Microbiome. 2017;5(1):1–13.

5. Zhao N, Chen J, Carroll IM, Ringel-Kulka T, Epstein MP, Zhou H, et al. Testing in microbiome-profiling studies with MiRKAT, the microbiome regression-based kernel association test. The American Journal of Human Genetics. 2015;96(5):797–807.
