# Supplementary material for: Testing microbiome associations with survival times at both the community and individual taxon levels
Source: PLoS Comput Biol. 2022 Sep 14;18(9):e1010509. doi: 10.1371/journal.pcbi.1010509 (PMC9512219; doi:10.1371/journal.pcbi.1010509)
Supplement: S1 Table — (PDF) [file pcbi.1010509.s002.pdf]

S1 Table. Type I error of the global tests for simulated data in other cases

				LDM-			permanovaFL-				
Censoring	n	Scenario	β_{XZ}	c	m	d	c	m	d	MiRKAT-S	OMiSA
75%	100	M1	0	0.050	0.049	0.053	0.051	0.051	0.052	0.051	0.053
			0.8	0.052	0.050	0.048	0.050	0.050	0.050	0.032	0.038
			0.8*	0.332	0.329	0.314	0.239	0.228	0.228	0.233	0.261
		M2	0	0.048	0.047	0.049	0.049	0.048	0.050	0.051	0.052
			0.8	0.051	0.053	0.049	0.050	0.050	0.047	0.009	0.038
			0.8*	0.469	0.480	0.442	0.475	0.473	0.437	0.474	0.261
25%	100	M1	0	0.052	0.051	0.052	0.052	0.051	0.051	0.053	0.052
			0.8	0.047	0.044	0.048	0.049	0.046	0.049	0.030	0.033
			0.8*	0.806	0.812	0.725	0.624	0.628	0.593	0.643	0.697
		M2	0	0.043	0.045	0.044	0.045	0.044	0.045	0.047	0.052
			0.8	0.049	0.047	0.046	0.049	0.050	0.047	0.009	0.033
			0.8*	0.912	0.919	0.86	0.925	0.931	0.864	0.931	0.694
50%	50	M1	0	0.045	0.046	0.044	0.045	0.046	0.044	0.051	0.047
			0.8	0.045	0.047	0.047	0.049	0.050	0.048	0.032	0.027
			0.8*	0.335	0.327	0.294	0.218	0.217	0.207	0.225	0.304
		M2	0	0.042	0.044	0.043	0.047	0.046	0.046	0.050	0.047
			0.8	0.044	0.045	0.040	0.046	0.047	0.044	0.006	0.027
			0.8*	0.462	0.471	0.435	0.474	0.477	0.445	0.489	0.304

Note: See the note to Table 1. All event times here were simulated from the Cox model.
