# Supplementary material for: Testing microbiome associations with survival times at both the community and individual taxon levels
Source: PLoS Comput Biol. 2022 Sep 14;18(9):e1010509. doi: 10.1371/journal.pcbi.1010509 (PMC9512219; doi:10.1371/journal.pcbi.1010509)
Supplement: S2 Fig — Left column: data were simulated and analyzed based on the relative abundance scale, same as in model M1. Right column: data were simulated and analyzed based on the presence-absence scale (except for OMiSA), same as in model M2. The censoring rate was 50% and n = 100. Results of sensitivity and empirical FDR were obtained when Xi was a confounder (βXZ = 0.8). (PDF) [file pcbi.1010509.s004.pdf]

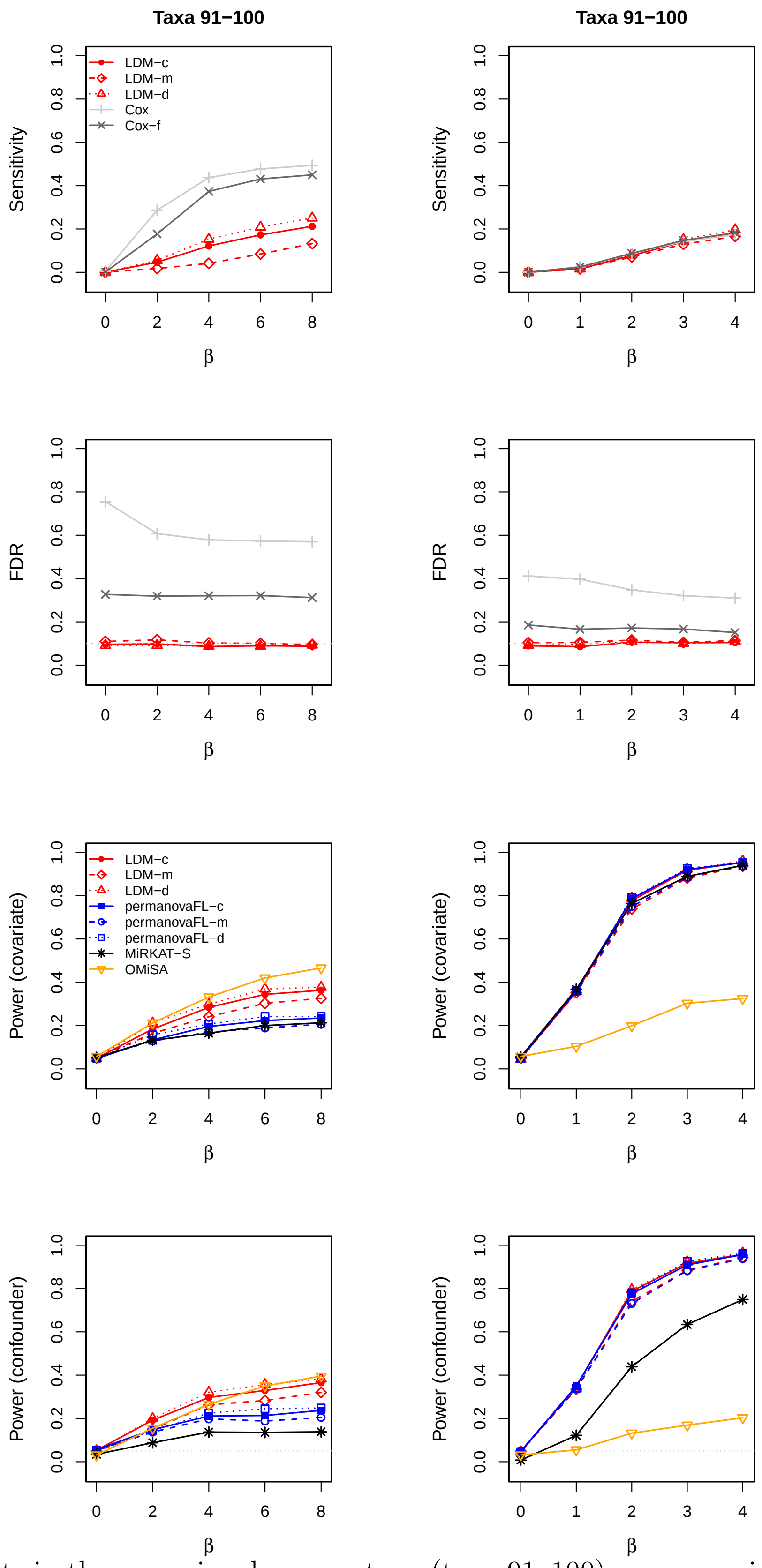

S2 Fig. Results in the scenario when rare taxa (taxa 91–100) were associated with the event time. Left column: data were simulated and analyzed based on the relative abundance scale, same as in model M1. Right column: data were simulated and analyzed based on the presence-absence scale (except for OMiSA), same as in model M2. The censoring rate was 50% and $n = 100$. Results of sensitivity and empirical FDR were obtained when X_i was a confounder ($\beta_{XZ} = 0.8$).
