# Supplementary material for: Testing microbiome associations with survival times at both the community and individual taxon levels
Source: PLoS Comput Biol. 2022 Sep 14;18(9):e1010509. doi: 10.1371/journal.pcbi.1010509 (PMC9512219; doi:10.1371/journal.pcbi.1010509)
Supplement: S4 Fig — Results of sensitivity and empirical FDR were obtained when Xi was a confounder (βXZ = 0.8). (PDF) [file pcbi.1010509.s006.pdf]

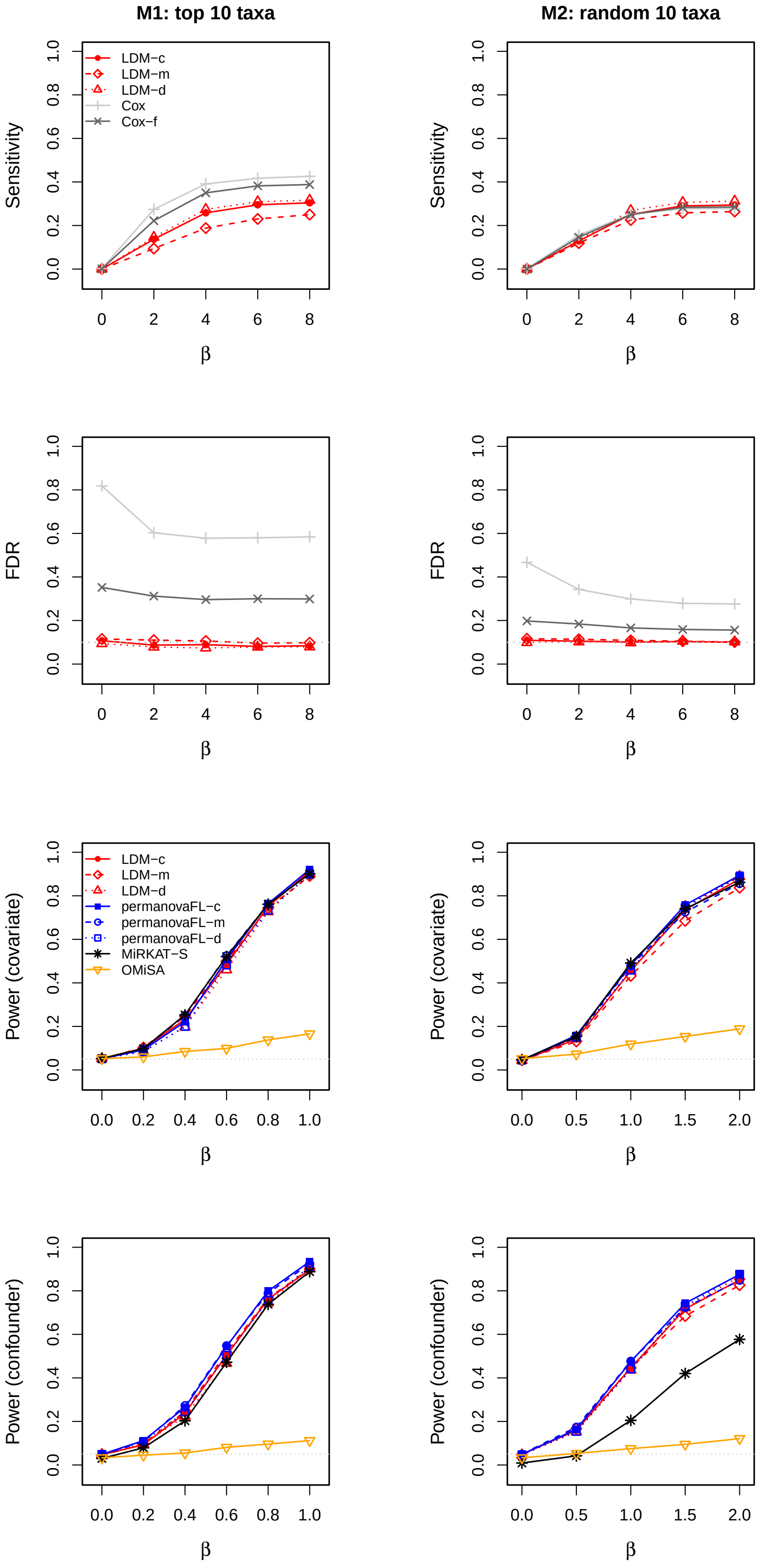

S4 Fig. Results for simulated data with 25% censoring and $n = 100$. Results of sensitivity and empirical FDR were obtained when X_i was a confounder ($\beta_{XZ} = 0.8$).
