# Supplementary material for: Testing microbiome associations with survival times at both the community and individual taxon levels
Source: PLoS Comput Biol. 2022 Sep 14;18(9):e1010509. doi: 10.1371/journal.pcbi.1010509 (PMC9512219; doi:10.1371/journal.pcbi.1010509)
Supplement: S6 Fig — The overdispersion parameter (“disp”) varied from 0.02, 0.002, to 0.0002. The results with overdispersion 0.02 are the same as those in Fig 1 (left column). (PDF) [file pcbi.1010509.s008.pdf]

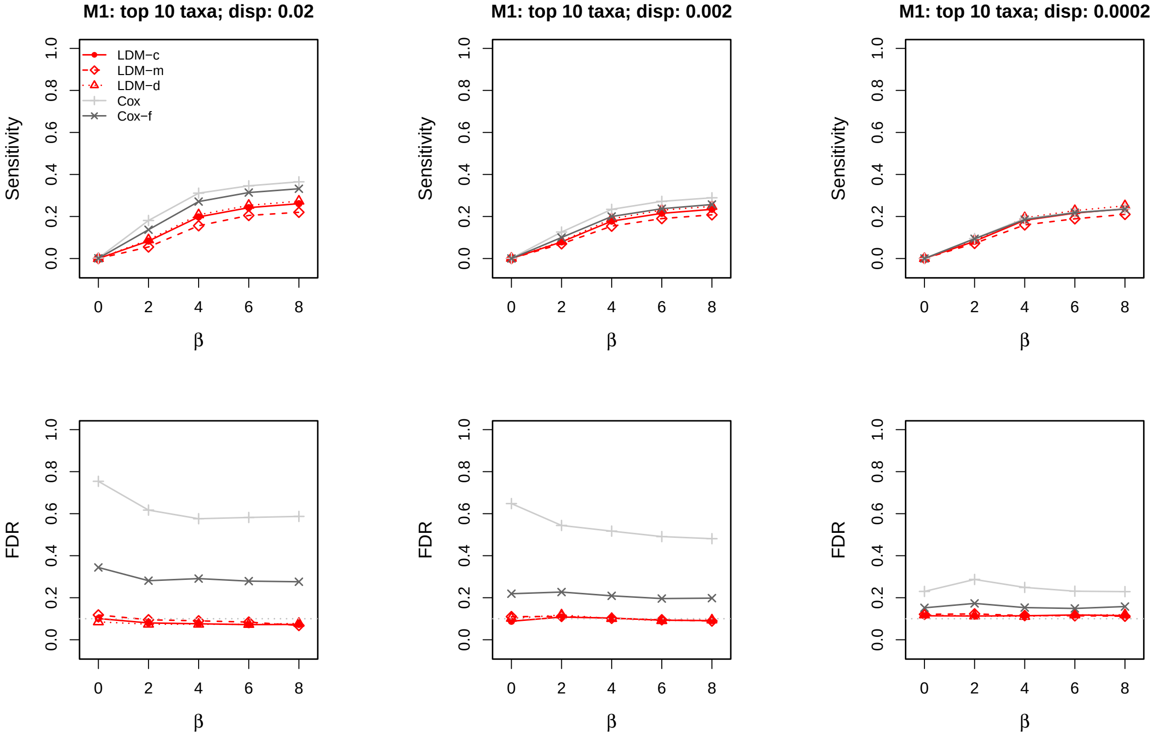

S6 Fig. Sensitivity and empirical FDR of the taxon-specific tests in analysis of simulated data with a confounder X_i ($\beta_{XZ} = 0.8$), 50% censoring, and $n = 100$. The overdispersion parameter ("disp"") varied from 0.02, 0.002, to 0.0002. The results with overdispersion 0.02 are the same as those in Fig 1 (left column).
