# Supplementary material for: Testing microbiome associations with survival times at both the community and individual taxon levels
Source: PLoS Comput Biol. 2022 Sep 14;18(9):e1010509. doi: 10.1371/journal.pcbi.1010509 (PMC9512219; doi:10.1371/journal.pcbi.1010509)
Supplement: S7 Fig — (PDF) [file pcbi.1010509.s009.pdf]

(a) Overall survival

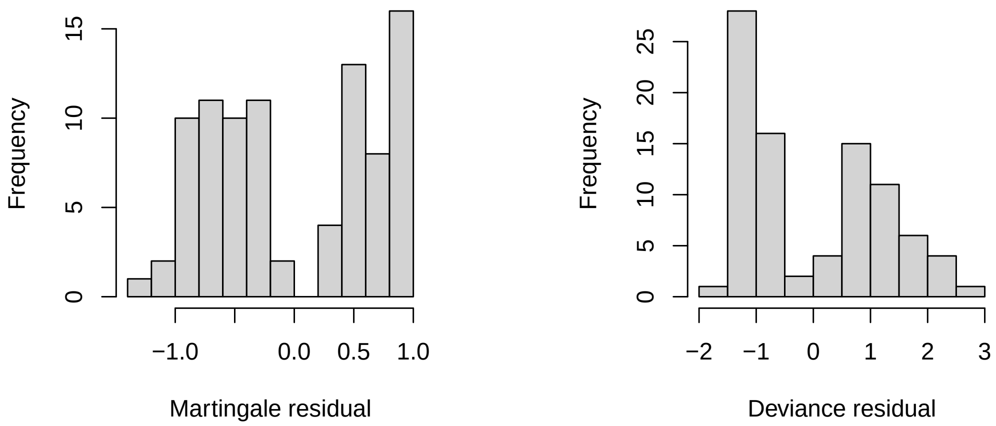

(b) Time to stage-III aGVHD

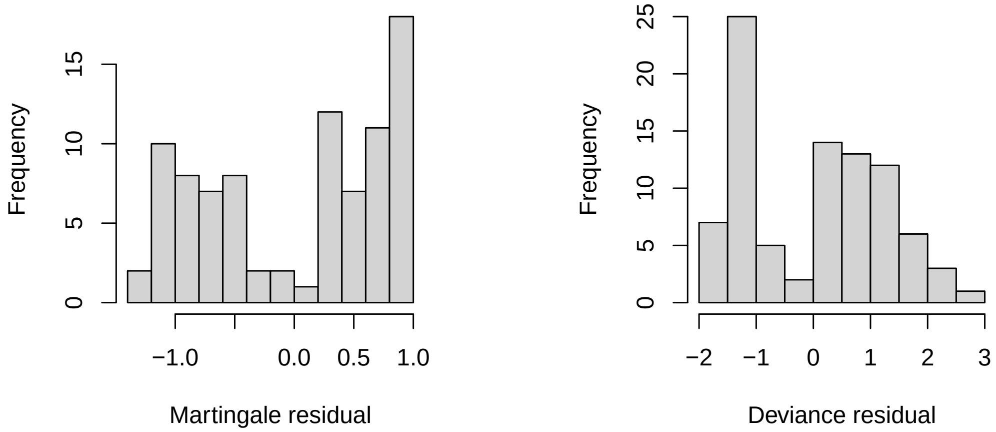

S7 Fig. Martingale and deviance residuals, generated from the Cox model that fit age and gender as covariates in analysis of the aGVHD data.
