# Supplementary material for: Testing microbiome associations with survival times at both the community and individual taxon levels
Source: PLoS Comput Biol. 2022 Sep 14;18(9):e1010509. doi: 10.1371/journal.pcbi.1010509 (PMC9512219; doi:10.1371/journal.pcbi.1010509)
Supplement: Attachment — Submitted filename: 2022-07-01_response.pdf [file pcbi.1010509.s010.pdf]

Response to Reviewer's Comments

Reviewer: 1

In this manuscript, Hu et al. extended their LDM framework to analyze survival outcomes. By treating the Martingale or deviance residuals as a covariate, their LDM framework can be conveniently applied. One nice thing about the proposed method is that it achieves both the community-level and individual taxa-level tests. Simulations and real data analysis demonstrated the superior performance of the proposed method to the ad hoc Cox model (individual taxa tests) and MiKRAT-S and OMiSA (global test). As clinical research has been increasingly adding the microbiome component, statistical methods for integrating the microbiome data into survival analysis are urgently needed. However, previous research mainly focused on community-level tests. Methods for individual taxa-level tests predominantly use the traditional Cox regression model by treating the taxon abundance as a covariate. It is not clear whether this method is valid or efficient. In this work, the authors revealed that the Cox model could not control the type I error properly, which is quite surprising. Overall, I believe the work is a nice contribution to the microbiome community. Once fully validated, I envision it to be an essential tool in statistical analyses of microbiome data. I have a couple of minor questions and comments that need the authors' feedback.

1. *I do not quite understand why the traditional Cox model has such a high FDR inflation. If the null model is generated according to the Cox model, I will expect the Cox model could control the type I error very well.*

   The high FDR inflation is due to the sparse non-zero data at many taxa. Sparse covariate data have been tied to inflated type I error using the Cox model [1, 2]. To confirm this hypothesis, we varied the overdispersion parameter from 0.02 to 0.002 and finally to 0.0002 to successively decrease the number of cells with zero counts. Indeed, as the data became less sparse, the FDR of both Cox models became less inflated. We have added these results to the new Figure S6 and described the results on page 12.

2. *The authors did not mention/address the potential compositional effects. For example, what if several highly abundant taxa are associated with survival in the same direction? I think the authors need to address it in the discussion by providing some possible solutions, e.g., using robust normalization (GMPR, CSS, etc.).*

   We have added the following paragraph to the Discussion section.

   In this article, we have primarily considered testing hypotheses that are expressed in terms of relative abundances. Some investigators may prefer to test hypotheses that are expressed in terms of ratios of counts or relative abundances. To this end, a common approach is to normalize the read count data using methods such as GMPR [3] and CSS [4] and then apply tests of differential abundance (such as the LDM) to the normalized

data. This approach critically depends on the validity of the normalization method of choice, which may not perform well in the presence of sparse read count data. In addition, the LDM can be directly applied to log-ratio data, although some of the appealing features of the LDM such as analyses on multiple scales must be suppressed.

3. *In the simulation, the 20% FDR cutoff may be too high. I think the authors should increase the signal strength instead of using a higher FDR since the real data uses 10% FDR.*

   We thank the reviewer for this helpful comment. We have rerun all simulation studies with nominal FDR 10%. All of our conclusions stayed unchanged.

4. *In the discussion, the authors mentioned the LDM-omni3. Could the authors provide the omni3 p-value for the real data?*

   We have added the LDM-omni3 p-values to Table 2. We have also added similar omnibus test results for other methods whenever possible. The omnibus test results tracked the results of the best-performing data scale in all cases. We described these omnibus tests and results on pages 15–16.

Reviewer: 2

In this manuscript, Hu and colleagues present a method for associating microbiome with censored survival times. They extend their previous method (and package, so that the method is available for others) to be able to both detect community associations as well as associating particular taxa with the outcomes. Overall, the methods appear to be well done and the benchmarks based on simulated data do show the advantages of this method. However, the application to real data shown in the current version is still limited. I think it would strengthen the manuscript to present more of the analyses of the aGVHD data in the main text. In particular, I recommend moving Fig S7 to a main figure. One important claim that the authors make is that applying the Cox model in an ad hoc model leads to unacceptably high false discovery rates. This is demonstrated in simulated data, but it would be important to see the effect in real data as well.

We thank the reviewer for these suggestions. We have now moved Figures S7–S8 to the main text to be Figures 4–5. We have also added the results of the Cox model and the Firth-corrected Cox model to Table 2.

Although not something that I would personally consider to be strictly required, adding some shotgun metagenomics data analyses would also further bolster the case that this method is widely applicable. In principle, it should be possible to apply the method; but shotgun data does have different error profiles.

We have added the following paragraph to the Discussion section.

"The gut microbiome data in the aGVHD dataset that we have analyzed here were generated from 16S rRNA sequencing. Our approach is readily applicable to microbiome data generated

from shotgun metagenomic sequencing, although these data have different error profiles than 16S rRNA sequencing data. In fact, in a recent publication [5], we have applied permanovaFL using the approach developed here to analyze the shotgun metagenomic sequencing data of the gut microbiome and the outcome data on progression-free survival that were generated from a melanoma immunotherapy study [6]."

Bibliography

[1] Chen H, Lumley T, Brody J, Heard-Costa NL, Fox CS, Cupples LA, et al. Sequence kernel association test for survival traits. Genetic epidemiology. 2014;38(3):191–197.

[2] Bi W, Fritsche LG, Mukherjee B, Kim S, Lee S. A fast and accurate method for genome-wide time-to-event data analysis and its application to UK biobank. The American Journal of Human Genetics. 2020;107(2):222–233.

[3] Chen L, Reeve J, Zhang L, Huang S, Wang X, Chen J. GMPR: A robust normalization method for zero-inflated count data with application to microbiome sequencing data. PeerJ. 2018;6:e4600.

[4] Paulson JN, Stine OC, Bravo HC, Pop M. Differential abundance analysis for microbial marker-gene surveys. Nature methods. 2013;10(12):1200–1202.

[5] Yue Y, Hu YJ. Extension of PERMANOVA to Testing the Mediation Effect of the Microbiome. Genes. 2022;13(6):940.

[6] Spencer CN, McQuade JL, Gopalakrishnan V, McCulloch JA, Vetizou M, Cogdill AP, et al. Dietary fiber and probiotics influence the gut microbiome and melanoma immunotherapy response. Science. 2021;374(6575):1632–1640.
