# Supplementary material for: Testing microbiome associations with survival times at both the community and individual taxon levels
Source: PLoS Comput Biol. 2022 Sep 14;18(9):e1010509. doi: 10.1371/journal.pcbi.1010509 (PMC9512219; doi:10.1371/journal.pcbi.1010509)
Supplement: Attachment — Submitted filename: 2022-08-04_response.pdf [file pcbi.1010509.s011.pdf]

Response to Reviewer's Comments

Reviewer: 2

However, unless I am mistaken, the new results in Table 2 (related to the results of the Cox model) actually show the opposite of what was claimed based on simulated data: the Cox model returns fewer positives (in several instances, zero taxa) than the alternatives. Thus, it becomes hard to see how this corresponds to unacceptably high false positive rates (rather than its opposite, a lack of statistical power). I feel that this point should be clarified before publication.

We thank the reviewer for identifying this discrepancy. We have added the following paragraph to the Discussion (page 18):

"In our simulation studies where the data were generated from the Cox model, we found that the tests based on the Cox model made many discoveries including excessive false discoveries, leading to inflated FDR. Conversely, in our analysis of the aGVHD data, we found that the Cox model made fewer discoveries and particularly zero discovery based on the relative abundance data. This disagreement reflects the fact that the aGVHD data do not follow the Cox model exactly. Indeed, some survival functions in Figures 4 and 5 showed violation of the proportional hazards assumption."
